# Log File Times as Indicators of Structured Figural Matrix Processing

**DOI:** 10.3390/jintelligence13060063

**Published:** 2025-05-28

**Authors:** Dominik Weber, Marco Koch, Frank M. Spinath, Florian Krieger, Nicolas Becker

**Affiliations:** 1 Department of Individual Differences & Psychodiagnostics, Saarland University, Campus A1 3, D-66123 Saarbrücken, Germany; marco.koch@uni-saarland.de (M.K.); f.spinath@mx.uni-saarland.de (F.M.S.); nicolas.becker@mx.uni-saarland.de (N.B.); 2 Department of Methods of Educational Research, TU Dortmund University, Emil-Figge-Straße 50, D-44227 Dortmund, Germany; florian.krieger@tu-dortmund.de

**Keywords:** figural matrices, log files, response time, time on task, item processing, goal management

## Abstract

Previous research has shown individual differences in (a) time on task (ToT) and (b) the degree of structuredness in processing figural matrices. The goal of this article was to integrate these two lines of research by analyzing log files from a computer-based assessment (*N* = 198) to examine the role of three ToT sub-components: onset times (before engaging with the first matrix rule), interrule times (between the rules), and intrarule times (within a single rule). We tested three clues that support the assumptions that the interrule times reflect the cognitive construction of a rule-specific solution plan, while the onset times represent a global orientation reaction, and the intrarule times capture the behavioral execution of the plan: (1) based on the interrule times, we identified two clusters of participants, of which one processed the matrices in a more structured fashion; (2) only the accelerating effect of the interrule times across the test was associated with test performance, indicating higher reasoning saturation; (3) a mediated path analysis revealed that faster interrule times propagate in faster intrarule times and more structured processing of matrix rules, resulting in better performance. Confirming internal validity, the three log file times accounted for an incremental 24.30% of test performance variance beyond the traditional ToT. Confirming external validity, two clusters were also identified based on the interrule times from the first test and performance scores from a second matrix test.

## 1. Introduction

Figural matrices are a widely used and economical method to assess reasoning ability (e.g., Marshalek et al. 1983; Raven et al. 1989). Previous research has shown that participants differ in the time spent on figural matrices (e.g., Becker et al. 2016; Goldhammer et al. 2015; Krämer et al. 2023) and the degree of structuredness when solving figural matrix tasks (e.g., Carpenter et al. 1990; Krieger et al. 2019; Weber et al. 2023). This article aims to integrate these two research strands by (a) determining finer response-time components of figural matrices processing, (b) examining to which extent these components provide information about the processing strategy, and (c) investigating their role in test performance.

### 1.1. Figural Matrices in Intelligence Diagnostics

**Figure 1:**
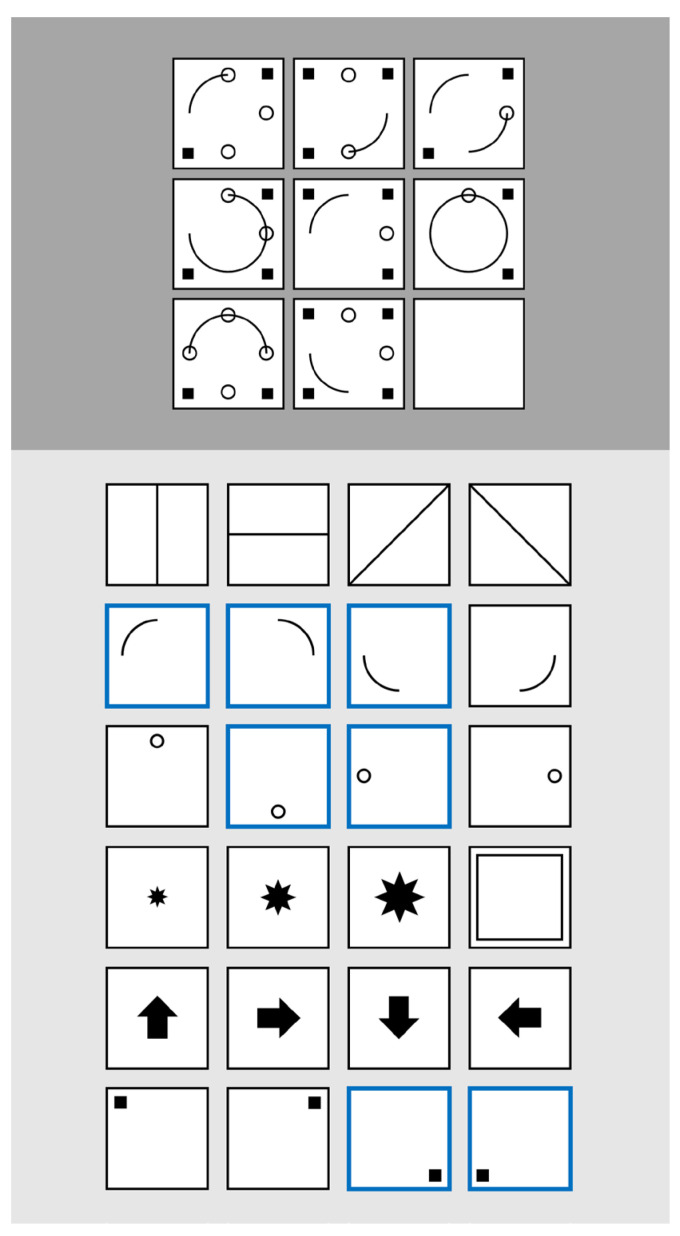
The figure shows a typical construction-based figural matrix. The item consists of an item stem (upper section) and a construction kit (lower section) to complete the item stem. The item row-wise contains three logical rules: addition (bent lines), subtraction (circles), and intersection (squares). The symbols that must be selected are highlighted in blue. Please note that this example is a modified item of the DESIGMA (Becker and Spinath 2014) to prevent leakage of the original items.

Figural matrices typically consist of a 3 × 3 grid of cells (e.g., Formann et al. 2011; Koch et al. 2022; Pallentin et al. 2023) containing geometrical symbols (e.g., lines, circles, squares). The arrangement of these symbols follows certain logical rules (e.g., *addition*: symbols in the first and second cell sum up in the third cell; *intersection*: only the symbols that appear in both the first and second cell are contained in the third cell). The last cell of the third row is empty, and the participants’ task is to complete this empty cell based on the rules in the rest of the matrix. To prevent participants from guessing the correct answer among distractors, distractor-free response formats have been invented: while some of these approaches require drawing the solution either manually (Mitchum and Kelley 2010) or by clicking pixels of the empty matrix cell (Piskernik 2013; cf. Breuer et al. 2020), several so-called construction-based matrix tests have been developed (e.g., Becker and Spinath 2014) that ask participants to compose the solution based on a construction-kit containing all possible symbols (Figure 1). To understand why figural matrices serve as such a strong indicator of reasoning ability, research particularly investigated the underlying processes of solving these kinds of items. Two lines of research have taken distinct explanatory approaches: One focuses on the temporal characteristics of matrices processing, while the other one examines whether participants vary in structuredness processing matrices.

### 1.2. Temporal Characteristics of Matrices Processing

Research has shown that participants differ in the amount of time they invest in solving an item. The total time that participants spend on an item is referred to as *Time on Task* (ToT). The *ToT effect* describes the change in the probability of solving an item as a function of the ToT. Goldhammer et al. (2015) found that a linear association between ToT and performance comes up short for figural matrices. Instead, they suggest that it is reasonable to differentiate the ToT effect depending on participants’ cognitive ability. Specifically, they found a negative ToT effect (i.e., the faster the item processing, the higher the performance) for more able participants, whereas less able participants exhibited a diminishing or slightly positive effect. In a comprehensive analysis, Krämer et al. (2023) replicated this pattern in nine of ten examined samples, revealing a stronger negative ToT effect for more able compared to less able participants.

Becker et al. (2016) noted that the findings of Goldhammer et al. (2015) were based on a sample with an above-average ability (i.e., only high school and university students). Similarly, Krämer et al. (2023) analyzed samples of applicants for the admission tests of German medical schools, who reported excellent school grades. In contrast, Becker et al. (2016) examined a more heterogeneous sample with diverse educational backgrounds. Their results confirmed the negative ToT effect for more able participants and also showed that this effect does not only diminish but becomes positive for less able participants. Overall, the relationship between ToT and performance appears to be quadratic. Goldhammer et al. (2014, 2015) provide a potential theoretical framework to explain this pattern. They propose a dual-processing theory of reaction times in a figural matrix: When a person’s ability exceeds the cognitive demands of a task, they process the task “automatically” (i.e., requiring sparse cognitive control, attention, and effort resulting in shorter ToT). In contrast, when a person’s ability falls short of the demands of a task, they must draw on cognitive control resulting in longer ToT.

However, while automatic processing appears plausible for learned information stored in the (long-term) memory (as originally proposed by Schneider and Shiffrin 1977) its application to figural matrix tasks remains challenging. Figural matrices require participants to identify complex relations between symbols (e.g., Robertson 2010) that occur across multiple matrices in varying combinations and positions with different underlying logical rules. Although it is conceivable that the logical principles (e.g., addition or intersection) become internalized over time, the specific content to which they must be applied varies from item to item, and the solution is therefore not immediately apparent. In other words, each matrix represents a novel problem making it (almost) impossible to store them in memory. Hence, it is reasonable to consider further sources of interindividual differences in matrix processing to account for the reported response time patterns.

### 1.3. Structuredness in Matrix Processing

Besides temporal differences, it has been shown that participants vary in structuredness when processing figural matrices. For instance, beyond simple *rule induction* Carpenter et al. (1990) proposed *goal management* as a key factor in solving figural matrices that decomposes a global goal (i.e., a figural matrix) into partial goals (i.e., the individual matrix rules). According to their two-process theory, goal management and rule induction are intertwined in a multiplicative matter, implicating that a failure in either process renders the item unsolvable (but see Preuß and Preckel 2025 who recently found evidence for a rather additive relationship, where each matrix rule appears to contribute independently to matrix difficulty). While, regarding goal management, the maintenance of the partial solutions does not seem to be the limiting factor of figural matrix performance, the ability to separate the global goal and to construct solutions for the partial goals was found to be crucial (Hayes et al. 2011; Krieger et al. 2019; Loesche et al. 2015).

Beyond the elimination of the guessing probability, construction-based matrix tests hold the potential for a deeper insight into item processing. While traditional distractor-based matrix tests only assess the response option selected by the participant (resulting in only one value per item), construction-based tests make it possible to track the sequence of symbols clicked within log files. From these fine-grained log files, it might be possible to infer the degree of structuredness in item processing. Chen et al. (2024) recently stated that the analysis of log files is becoming increasingly popular in contemporary research (see also Nicolay et al. 2023, who used log files to code individual strategies for complex problem-solving). For instance, log files were used to emphasize that participants with a higher reasoning ability process figural matrices in a more structured way (Weber et al. 2023). To this end, based on the log files of a construction-based matrix test it was computed how many times participants jumped between the matrix rules. A rule jump was defined as a click on a symbol of a specific rule (e.g., addition), followed by a click on a symbol of another rule (e.g., subtraction). The correlation between a rule jump score (executed rule jumps minus the rule jumps necessary for the correct solution) and the test performance was strongly negative.

### 1.4. Confluence of Temporal Characteristics and Structuredness in Matrix Processing

Beyond the analysis of rule jumping, log files make it possible to gain insight into the temporal characteristics of item processing. It is plausible that differences in the structuredness of item processing manifest in processing times. Log files can be used to go beyond the traditional global ToT by separating it into several components which might have different meanings for item processing and might allow inferences regarding the item processing strategy. In general, figural matrix processing can be separated into three temporal intervals which we call log file times (LFTs): (1) The time that elapses before participants start interacting with the first matrix rule (*onset time*); (2) The time that elapses between terminating one rule and beginning to process the next one (*interrule time*). For instance, if a matrix contains two rules (e.g., addition and subtraction) the interrule time refers to the interval between the clicks on the last symbol of the addition rule and the first symbol of the subtraction rule; (3) The time that elapses while processing a single rule (*intrarule time*), i.e., the interval between the click on a symbol of a specific rule and on a second symbol of the same rule. If an item consists of more than two rules and a rule of more than two symbols, several interrule and intrarule times per item are possible.

The aim of this research is to join two so far distinct lines of research by examining temporal characteristics of matrix processing in order to obtain a finer-grained insight into the processes involved. Previous research has found that a more structured item processing in terms of goal management is associated with higher performance (Hayes et al. 2011; Weber et al. 2023). Goal separation can be performed on two levels for figural matrices: (1) On the one hand, the separation of the total matrix into the symbol groups that are associated with different rules. This separation should be made immediately at the beginning of the item presentation and require only a few cognitive resources, as the symbol groups are easy to distinguish. Regarding the LFTs, the onset times (i.e., the times before the mental construction of the solution to the first rule) might be the temporal counterpart to this separation. (2) On the other hand, a separation can be performed regarding a specific rule by separating the corresponding symbol group into the symbols and identifying the underlying logical connections. The temporal counterpart to this might be the interrule times, in which a rule-specific mental plan for the next rule might be constructed. Finally, the intrarule times are likely to be the temporal counterpart to the execution of the constructed plan. Hence, we assume the LFTs to reflect three processes varying in demands on participants’ cognitive resources: The interrule times are hypothesized to reflect the core cognitive component of matrix processing with high cognitive demands. The onset times might reflect a cognitive orientation component with lower cognitive demands. And, the intrarule times might reflect a behavioral component of matrix processing with small cognitive demands.

### 1.5. Research Questions and Hypotheses

To test these assumptions, we first asked whether the LFTs can provide a deeper insight into the item processing strategy (Research Question 1; RQ 1). Therefore, after a replication hypothesis on the ToT effect (H1), we tested three hypotheses to find three clues that the interrule times reflect the core cognitive component of matrices processing (H2–H4):

**H1.**  *We assumed to confirm the ToT effect for figural matrices i.e., to find a quadratic association between the performance and the ToT. Participants with a medium performance were expected to spend most time on the items while participants with low and high performance were expected to spend less time.*

**H2.**  *We expected to find two clusters of participants with different processing strategies based on the interrule times and test performance: one cluster that applies rule-specific goal management (structured cluster) and one cluster that does not apply rule-specific goal management (unstructured cluster). To confirm this interpretation of the two clusters, we assessed whether the participants of the structured cluster (a) jump less frequently between different rules and (b) invest more time between the rules since they construct processing plans before solving the rules whereas the unstructured cluster tend to start processing immediately with less elaborated strategies.*

**H3.**  *In terms of a training effect, the LFTs should diminish throughout the matrices test. If the interrule times reflect the cognitive component of matrix processing its accelerating effect across the items of a test should correlate with the test performance in the structured cluster. We derived this hypothesis from the observation that individuals with higher cognitive ability tend to benefit more from cognitive tasks (i.e., basic cognitive ability predicts performance improvement over time; e.g., Ørskov et al. 2021; Wiemers et al. 2018). In contrast, the accelerating effects of the onset and intrarule time, as well as the accelerating effect of the interrule time in the unstructured cluster, should not be performance-associated.*

**H4.**  *In the case of a structured processing strategy, we assumed the interrule times to reflect the part of matrix processing in which participants construct a mental solution to a matrix rule. As a sign of higher cognitive abilities, faster interrule times might be associated with a better mental plan of how to construct the solution which might facilitate executing this plan. This might result on the one hand in faster intrarule times and on the other hand in fewer rule jumps. Hence, we expected the correlation between the interrule times and the test performance to be mediated by the intrarule times and the rule jumps.*

Second, we asked what role the LFTs play in test performance (Research Question 2; RQ 2). In this regard, we had the following hypothesis:

**H5.**  *Focusing on the structured cluster, we expected shorter LFTs to be associated with higher performance. Since the LFT contains more information about the item processing they should explain performance variance beyond the traditional ToT.*

Our hypotheses were formulated a priori to the analyses. However, we did not preregister the study, as it was based on existing data. In subsequent post hoc analyses, we examined whether the validity of the interrule times for clustering participants regarding their processing strategy could be extended to a second matrix test. To disentangle the dual role of the first matrix test score as both the clustering variable and the criterion, we conducted an additional cluster analysis using the interrule times from the first test and the scores from a second matrix test. If it is possible to replicate the clustering based on an independent intelligence test resulting in a pattern analogous to the original test-based scores, this would provide further support for the cognitive reflection of the interrule times. In addition, we post hoc explored indicators of potentially interfering motivational influences in matrix processing.

## 2. Materials and Methods

### 2.1. Sample and Materials

Our sample consisted of *N* = 198 participants who were assessed for monetary compensation. They had a mean age of *M* = 23.00 (*SD* = 4.43) years, and 75.25% of the participants were female. After an instruction item, they processed a computer-based version of the Design a Matrix test (DESIGMA; Becker and Spinath 2014). As a construction-based figural matrix test, the participants are not provided with response options to an item, but they must compose the response by means of a construction kit. They can select out of six symbol groups (e.g., quarter circles) with four symbols each (e.g., quarter circle on the left and the right top, the left and the right bottom) by clicking (please see Figure 1 for the principles of construction-based matrices). Each symbol group is associated with one of seven rules (addition, subtraction, disjunctive union, intersection, rotation, resizing, completeness) that participants must identify and apply to the last cell of the matrix. The maximum time participants could spend on an item was 90 s, which is a relatively mild time constraint (see the mean ToT in the descriptive statistics section) and might be a better alternative to a global test time constraint, which has been shown to be associated with skipping items and poorer strategy use in figural matrices (e.g., Gonthier 2023). Since single rule items require no rule jumps, they do not provide any information about the structuredness of the item processing. Hence, we only analyzed items with two or more rules. The final item set consisted of 22 items (eleven items with two rules, seven with three rules, three with four rules, and one item with five rules).

Furthermore, we assessed the participants with Raven’s Advanced Progressive Matrices (RAPM) Set II (Raven et al. 1988) as a traditional matrix test. In contrast to the DESIGMA, the RAPM is a distractor-based matrix test with eight response options per item. The participants randomly either processed the RAPM full-item set of 36 items (*n* = 94) or a short version of 24 items (*n* = 103; *n* = 1 participant only took part in the DESIGMA). The items of the short version were selected to maintain the same distribution of matrix rules as in the full-item version.

### 2.2. Statistical Analyses

As a performance measure, we computed the total score over the 22 items. As predictors, we computed from the log files of the assessment the mean ToT on the one hand and the mean LFTs (onset times, interrule times, intrarule times) on the other hand. Since the time between the start of the item presentation and the first click also contains the first rule-specific time, we computed the onset times as the residuals from regressing the rule-specific interrule times on the time before the first click. Further, analogously to Weber et al. (2023) we computed the mean difference between the applied and the necessary number of rule jumps.

To replicate the ToT effect that for participants with lower ability a higher ToT and for participants with higher ability a lower ToT is more effective, we tested for a quadratic regression and compared it with the linear model. We ran a cluster analysis based on the standardized test performance and the standardized interrule times as a supposed indicator of whether participants applied a structured processing strategy in terms of goal management (i.e., they construct rule-specific plans to the item rules as partial goals). The analysis was performed using hierarchical agglomerative clustering (Ward’s method with Euclidean distance), with 100 bootstrap iterations, evaluating cluster solutions ranging from *k* = 1 to *k* = 10.

To analyze accelerating effects we computed the mean times in the first third of the test and compared them to the mean times in the last third. To underline that the interrule times reflect the cognitive component of item processing critical for the test performance we regressed the performance on the accelerating effects. To obtain a better insight into the role and the interplay of the interrule and intrarule times we ran a path analysis with parallel mediation effects of the intrarule times and the rule jumps on the correlation between the interrule times and the test performance. We performed bootstrapping with 1000 iterations.

To examine whether in the structured cluster the speed (a) to obtain a rough overview at the beginning of the items, (b) to construct a rule-specific mental processing plan, and (c) to execute the constructed plan goes along with higher performance, we computed the correlations between the test score and the (a) onset, (b) interrule and (c) intrarule times. To prove whether it is worth considering the LFTs beyond the traditional ToT we ran a hierarchical regression.

To disentangle the dual role of the DESIGMA score as both the clustering variable and the criterion, we conducted an additional cluster analysis with the RAPM score—for both the subsamples that were assessed with the RAPM full-item version and the short version, respectively. We examined whether the interrule times, as the supposed temporal counterpart of cognitive ability, could account for incremental variance of the DESIGMA score beyond the RAPM score.

As a post hoc analysis, we sought indicators of potentially interfering motivational effects. To this end, we considered two approaches: (1) response to difficulty (RtD) and (2) incomplete attempt propensity (IAP). The RtD approach was derived from Cheyette and Piantadosi (2024), who found that the willingness to devote more time to more difficult items accounted for up to 42% of the variance in Raven’s Progressive Matrices (Raven et al. 1989). For each participant, we computed the RtD as the slope of an intraindividual regression of item response time on item difficulty. Higher slopes indicate a greater willingness to invest more time in solving more difficult items. We then controlled the RtD in a multiple regression predicting test performance by the interrule times. The IAP was derived from our log data and reflects a participant’s tendency to terminate an item without attempting all the rules of a matrix that are necessary for the correct solution. We computed a linear mixed-effect model including two components: (a) an individual’s global IAP, calculated as the individual’s mean IAP across the 22 items, and (b) a within-person (current) IAP, calculated by group-mean centering the individual’s IAP for each item, potentially indicating occasional lack of effort. Both global and current IAP were used to predict response time, controlling for participant and item effects. Finally, we determined whether controlling for global IAP affects the prediction of test performance by the interrule times.

We conducted our statistical analysis in *R* (R Core Team 2024). For data preparation, we used the packages *dplyr* (Wickham et al. 2023) and *stringr* (Wickham 2019); for data analysis, the packages *car* (Fox and Weisberg 2018), *effsize* (Torchiano 2020), *jmv* (Selker et al. 2022), *lavaan* (Rosseel 2012), *lm.beta* (Behrendt 2023), *lmerTest* (Kuznetsova et al. 2017), *MASS* (Venables and Ripley 2002), and *psych* (Revelle 2022); and for visualization, the packages *factoextra* (Kassambara and Mundt 2020) and *ggplot2* (Wickham 2016).

## 3. Results

### 3.1. Descriptive Statistics

Participants solved on average *M_score_* = 7.89 (*SD_score_* = 6.02) of the 22 items. The mean item difficulty was *P* = .36 and the mean corrected item-total correlation *r_i_*_(*t-i*)_ = .55. The internal consistency was very high at α = .92. On average, participants spent *M_ToT_* = 51.79 s (*SD_ToT_*=14.29 s) per item, of which *M_onset_* = 20.96 s (*SD_onset_* = 8.16 s) were for the onset time, *M_inter_* = 13.35 s (*SD_inter_* = 5.95 s) per rule for the interrule time and *M_intra_* = 1.42 s (*SD_intra_*=0.71 s) per click for the intrarule time. Internal consistencies were α_ToT_ = .93, α_onset_ = .89, α_inter_ = .87, and α_intra_ = .65. Table 1 shows the item characteristics of the 22 matrices items.

### 3.2. Replication of the ToT Effect

**Figure 2:**
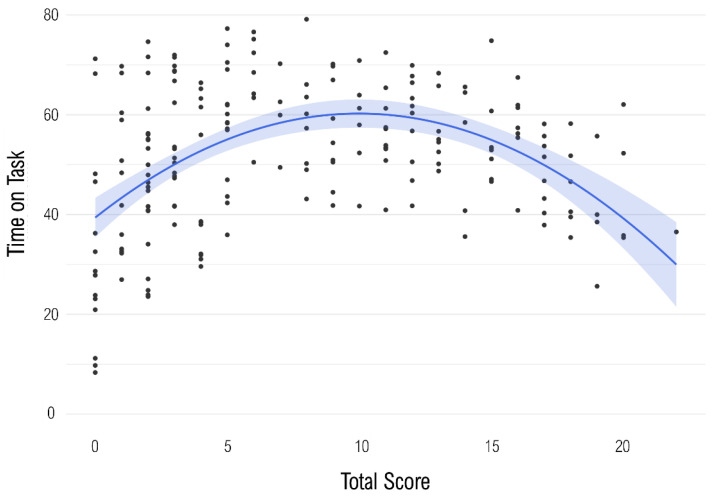
The figure shows the quadratic relationship between the DESIGMA score and the mean time on task per participant.

The total test score and the ToT showed a significant quadratic association: β = −1.68, *p* < .001. The quadratic model accounted for *R*^2^ = 22.78% of the variance, which were incremental Δ*R*^2^ = 20.88% compared to the linear model: *F*(1, 195) = 52.70, *p* < .001. Figure 2 shows the relationship between the two variables.

### 3.3. Clustering

**Figure 3:**
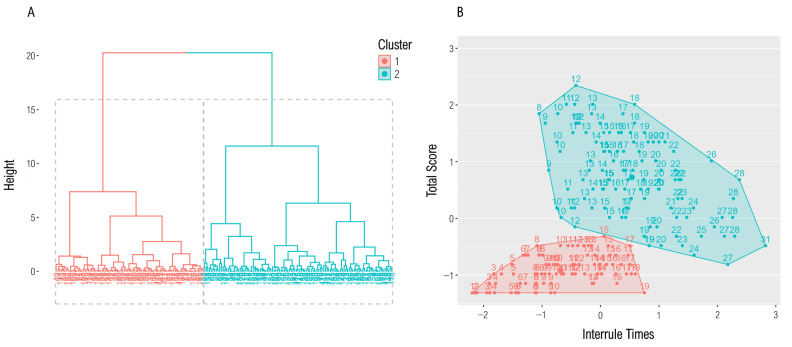
The figure shows the results of a cluster analysis based on the interrule times and the total test score. The analysis resulted in two clusters: cluster 1 consisted of *n* = 87, cluster 2 of *n* = 111 participants. (**A**) Dendrogram from the analysis; (**B**) clustered scatter plot: the variables have been scaled, while the (rounded) raw interrule times are displayed in the diagram.

The hierarchical agglomerative cluster analysis based on the interrule times as a supposed indicator of whether participants applied a structured processing strategy resulted in two clusters with *n*_1_ = 87 and *n*_2_ = 111, respectively (Figure 3). We determined the optimal number of clusters based on the dendrogram and the silhouette width which was highest for the two-cluster solution (average silhouette width = 0.47). Table 2 shows the mean test scores, the mean ToT, and the mean LFT per cluster. Cluster 2 achieved higher test scores than cluster 1 [*t*(146.15) = 21.90, *p* < .001, Cohen’s *d* = 2.86] and invested more time between the rules than cluster 1 [*t*(196) = 10.77, *p* < .001, Cohen’s *d* = 1.54] which might indicate the elaboration of a processing plan before the rules in cluster 2. Further, participants of cluster 2 jumped less frequently between the rules of an item: *t*(157.27) = −4.94, *p* < .001, Cohen’s *d* = −0.73. Hence, for the following analyses, we declared cluster 1 as the unstructured and cluster 2 as the structured cluster. We interpreted this finding as a first clue for the assumption that the interrule times reflect the cognitive component of matrix processing.

### 3.4. Accelerating Effect of the Log File Times

As expected, in both clusters, the LFTs diminished across the test, indicating an accelerating effect (i.e., participants spent less time on the items as they moved through the test; Table 3). The strongest acceleration was observed in the onset times, suggesting that participants adapted more quickly to new items later in the test compared to earlier ones. However, only the acceleration of the interrule times correlated with the DESIGMA test performance: *r* = .31 (95% CI [.13, .47]), *p* < .001. This finding is in line with previous research showing that task improvement is correlated with basic cognitive ability (e.g., Ørskov et al. 2021; Wiemers et al. 2018). We interpreted this finding as a second clue that in contrast to the onset and intrarule time, the interrule time has a stronger reasoning saturation and reflects the cognitive component in matrix processing.

### 3.5. Interplay of the Interrule and Intrarule Times

**Figure 4:**
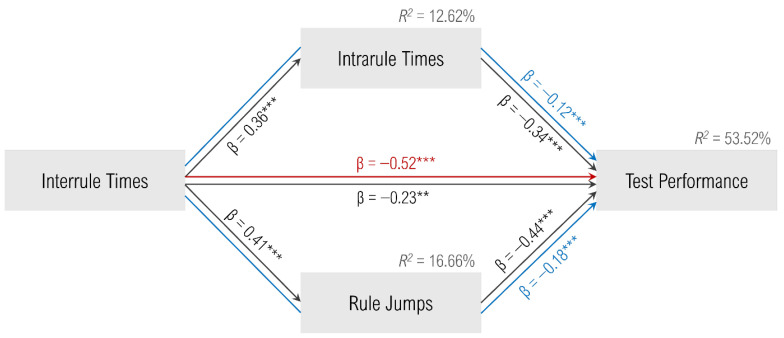
Path model with the results of the parallel mediation analysis; red path = total effect, black paths = direct effects, blue paths = indirect (mediated) effects, R^2^ = coefficient of determination, ** *p* < .01, *** *p* < .001.

In the following, we analyzed the importance of the LFTs for structured item processing (i.e., in the structured cluster). The path analysis to gain a deeper understanding of the roles of the interrule and intrarule times resulted in a parallel mediation model with a good fit: χ^2^(1) = 3.05, *p* = .081; CFI = .983, RMSEA = .136, *p*(RMSEA ≤ .050) = .123, SRMR = .052. Both the direct effect of the interrule times on test performance (β = −0.23, *p* = .001) and the two indirect effects were significant: interrule times on test performance via intrarule times (β = −0.12, *p* < .001) as well as via the number of rule jumps (β = −0.18, *p* < .001). Hence, we concluded an incomplete mediation. The model is demonstrated in Figure 4. We interpreted these mediation effects as a third clue for the cognitive reflection by the interrule times.

### 3.6. Importance of the Log File Times for Test Performance

The onset times in which participants might obtain a rough overview of the item, and the test score were negatively correlated, *r* = −.40 (95% CI [−.55, −.23], *p* < .001), i.e., participants with a higher performance needed less time to start processing the item. Further, the interrule times in which participants might construct a rule-specific processing plan had a strong negative correlation with the test score, *r* = −.51 (95% CI [−.64, −.36], *p* < .001), i.e., higher test performance goes along with less time between the rules. Finally, there was a strong negative correlation between the intrarule times in which participants might execute their rule-specific processing plan and the test score, *r* = −.53 (95% CI [−.65, −.38], *p* < .001), i.e., higher performant participants executed the processing plans more quickly.

In order to determine whether the LFT has an incremental value to the test performance beyond the traditional ToT we computed a hierarchical regression (Table 4). The overall model with all response times (ToT and LFT) accounted for *R*^2^ = 56.00% of the test score variance, *F*(4, 106) = 33.73, *p* < .001. Since the VIFs of the response times were below the critical threshold of VIF = 10 (VIF_ToT_ = 5.51, VIF_onset_ = 2.16, VIF_inter_ = 3.91, VIF_intra_=1.27), there was no multicollinearity of the response times. Each response time contributed significantly to the prediction of the test performance. The LFT explained incrementally Δ*R*^2^ = 24.30% beyond the ToT, *F*(3, 107) = 19.52, *p* < .001.

### 3.7. External Validation of the Interrule Times

**Figure 5:**
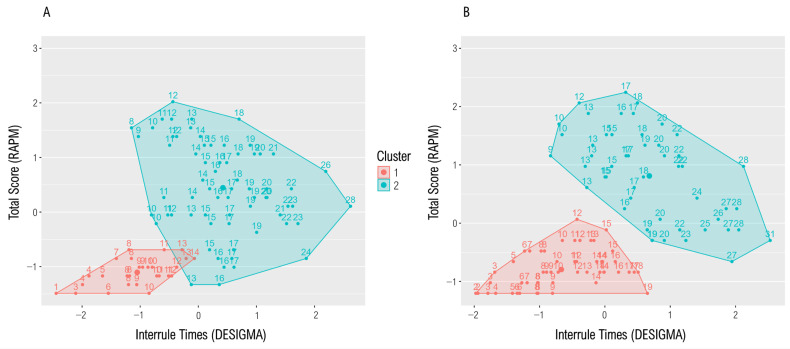
Results of the cluster analysis based on the interrule times from the DESIGMA and the RAPM score; (**A**) based on the RAPM full-item version, two plausible clusters of the sizes *n_1_* = 27 and *n_2_* = 67 could be identified; (**B**) also based on the RAPM short version, two plausible clusters of the sizes *n_1_* = 52 and *n_2_* = 51 could be identified.

On average, participants solved *M* = 24.27 (*SD* = 6.33) of the 36 RAPM full-item version items and *M* = 14.92 (*SD* = 4.67) of the 24 RAPM short version items. Internal consistencies were good with α = .87 and α = .84, respectively. For both RAPM versions, two plausible clusters based on the interrule times of the DESIGMA could be identified, showing a similar pattern to the clusters derived from the DESIGMA score (Figure 5). One cluster processed the matrices in a more structured manner with fewer rule jumps than the other one: (full-item version) *t*(48.62) = −2.26, *p* = .014, Cohen’s *d* = −0.51; (short version) *t*(88.57) = −3.60, *p* < .001, Cohen’s *d* = −0.71. In the structured cluster of both RAPM version, the interrule times accounted for incremental 6.21% (full-item version) and 22.42% (short version) of the DESIGMA score variance beyond the RAPM score, which was a significant model improvement due to the inclusion of the interrule times for both subsamples: (full-item version) *F*(1, 64) = 5.88, *p* = .018, (short version) *F*(1, 48) = 18.97, *p* < .001.

### 3.8. Post-Hoc Analyses of Potential Task Misunderstanding and Motivational Effects

The cluster which we called “unstructured” consisted of *n* = 87 participants representing 43.94% of the total sample. On average, these participants solved only 2.34 of the 22 DESIGMA items. Notably, 15 participants did not solve any item. To rule out the possibility that this poor performance was due to a misunderstanding of the matrix task, we analyzed the responses of these 15 participants to the DESIGMA items with only one logical rule, which had been excluded from the primary analyses. Actually, the 15 participants solved an average of 3.60 (*SD* = 0.91) out of the four one-rule items. In addition, they solved an average of 14.20 (*SD* = 7.37) rules across the 22 multi-rule items. Since guessing the correct answer in a construction-based matrix test is highly improbable, this response pattern suggests that the participants indeed understood the task but failed to apply goal management just as they had to handle multiple rules. This interpretation is supported by the finding that this cluster exhibited significantly more rule jumping compared to the structured cluster.

The slopes of the RtD effect, as an indicator for motivated processing, were on average *M*_β_ = 0.23 (*SD*_β_ = 0.25), indicating that participants tended to spend more time on more difficult items. RtD accounted for *R*^2^ = 16.96% (β = 0.38, *p* < .001) of the test performance variance, and for *R*^2^ = 6.68% (β = 0.26, *p* < .001) and *R*^2^ = 12.28% (β = 0.35, *p* < .001) of the ToT and interrule times variance, respectively. However, in a multiple regression including RtD as an additional predictor, the interrule times remained a strong predictor of test performance (β = −.51, *p* < .001).

A total of 16.09% of participants in the unstructured and 19.82% in the structured cluster did not attempt at least one item. However, only two participants skipped more than five of the 22 DESIGMA items. The global IAP, representing the general tendency to not attempt solving all rules of an item, was slightly higher in the unstructured cluster [*t*(166.59) = −2.07, *p* = .040], but not associated with test performance (β = 0.12, *p* = .084). The global IAP predicted the ToT, but not the interrule times (Table 5), suggesting that the interrule times are more robust against motivational differences across participants. The current IAP, used as an indicator for intraindividual variation in effort, did not predict either the ToT or the interrule times (Table 5), indicating that these two temporal measures are not affected by occasional lack of effort. In a multiple regression including the global IAP, the interrule times remained a strong performance predictor (β = −0.53, *p* < .001).

## 4. Discussion

### 4.1. Summary and Contextualization of the Results

The goal of this article was to combine two lines of research on figural matrix processing: previous research has shown that structured item processing is associated with a higher test performance (Weber et al. 2023). In this regard, goal management is a crucial factor (Carpenter et al. 1990), especially the capacity to separate the matrix into subgoals and to construct solutions to them (Krieger et al. 2019; Loesche et al. 2015). Furthermore, it has been shown that for more able persons a shorter ToT goes along with higher performance in matrix tests (Becker et al. 2016; Goldhammer et al. 2015; Krämer et al. 2023). Based on this research, we used reaction times from log files of a computer-based matrix test to draw conclusions regarding matrix processing. We separated the traditional ToT into three LFT components: (1) the onset times before interacting with the first matrix rule, (2) the interrule times between two rules, and (3) the intrarule times within a single rule.

Addressing RQ 1 to which extent the LFTs can provide a deeper insight into matrix processing, VIF analysis showed no multicollinearity of the LFTs, indicating that they are three distinct temporal measures. We found three clues that the interrule times reflect the core cognitive component of matrix processing in which participants construct a mental solution plan to a matrix rule: First, based on the interrule times and the test performance, we identified two clusters of participants. One of the clusters spent more time between the rules (which might indicate a stronger elaboration of a mental solution plan) and jumped less between rules (i.e., applied more structured processing) than the other cluster. Second, only the accelerating effect of the interrule times but not that of the onset or intrarule times was associated with the test performance, indicating a higher reasoning saturation. Third, within a mediated path model faster interrule times propagated on the one hand in faster intrarule times and, on the other hand, in fewer rule jumps, both resulting in better test performance. Hence, we conclude that the onset times reflect a global orientation process with rather low cognitive demands, that the interrule times are the temporal counterpart of the participant’s construction of a mental solution plan with high cognitive demands, and that during the intrarule times, participants execute this plan with behavioral demands. These findings support the proposal of Carpenter et al. (1990) that goal management is a crucial component of matrix processing and extend the understanding of structured matrix processing.

Addressing RQ 2, whether the LFTs are a valid measure compared to the traditional ToT, we found that they accounted incrementally for 24.30% of the performance variance when applying structured matrix processing, with each LFT contributing uniquely. The strong predictive effect of the interrule times on test performance was not affected by motivational factors. To abstract from the matrix test from which the LFTs were derived, we performed additional cluster analyses based on the scores of a second, independent matrix test. In two subsamples, the interrule times again successfully differentiated participants into two clusters, analogously to the test-inherent clusters.

### 4.2. Limitations and Future Perspectives

First, it should be noted that the findings of this study are based on behavioral processing patterns, and that the conclusions regarding participants’ strategies are implicit. We chose this approach to evaluate the potential of log data, which Chen et al. (2024) recently emphasized in their literature review as an auspicious lever for enhancing psychological assessments. Since log files are generated automatically in many computerized intelligence tests the consideration of the LFTs in diagnostics could be a very economical way to obtain more fine-grained test results. Alternative approaches for future research might include the use of think-aloud protocols or eye-tracking methodologies.

A second limitation is the borderline internal consistency of the intrarule times (α = .65). A plausible explanation for this might lie in the generally low values of the intrarule times (*M* = 1.42). Minor variations in an individual’s clicking behavior (e.g., whether participants kept their hand on the mouse or not) may have had a comparatively large impact on these low values. In contrast, the internal consistencies of the onset (α = .92) and interrule times (α = .87) were high, which is important for this study since we identified the interrule times in particular as the temporal counterpart of cognitive ability. Notably, their internal consistency was comparable to that of the test score which supports their potential use as an additional diagnostic indicator in intelligence diagnostics.

A third point that warrants discussion is the potential role of motivation or effort. To investigate this, we examined the effects of two indicators of motivation: the RtD, which we adopted from the literature (Cheyette and Piantadosi 2024), and the IAP, which we derived from our log data. Although both indicators were correlated with either the test performance or temporal measures, controlling for them did not reduce the strong predictive effect of the interrule times on test performance. This suggests that the interrule times are largely unaffected by motivational factors. An advantage of these two indicators is that they are based on test-inherent processing behavior, making them highly naturalistic. However, they are implicit measures. To conclusively rule out motivational effects, future research could complement this approach with self-report measures of motivation or effort administered alongside the matrix items.

Our research provides evidence that cognitive ability and distinct processing strategies are reflected in response times on figural matrices. However, this does not imply that interindividual differences in response times are fully explained by cognitive parameters alone. In addition to motivational factors, differences in personality traits may also contribute to variation in response times. For instance, conscientiousness and achievement motive might influence (1) whether a participant develops a processing plan at the beginning of an item, (2) how much effort and time they invest when having difficulty solving the item (potentially leading to longer response times for incorrectly answered items compared to correctly solved ones), and (3) the tendency to double-check or assure a solution (possibly resulting in more time between the last click on a symbol of the construction-kit and the submission of the response). Future research could examine these personality traits to further elucidate why individuals differ so markedly in both performance and response times on figural matrices.

## 5. Conclusions

Our research enables a finer-grained differentiation of the traditional ToT by introducing log file-based reaction times and shows initial evidence of the relevance of these LFTs for item processing and the test performance on figural matrix tests. Our findings support the assumption that the onset times at the beginning of matrix processing reflect a global orientation process, that the interrule times reflect the cognitive construction of a mental solution plan, and that the intrarule times reflect the execution of this plan. The LFTs account for incremental test performance variance beyond the traditional ToT. These findings lead to the recommendation to track response times of computer-based assessments by log files when feasible and to consider them within research on item processing strategies and on the ToT effect.

## Figures and Tables

**Table 1:** Item characteristics of the figural matrices.

Item	Rules	*P*	*r_i_*_(*t-i*)_	*M_ToT_*	*M_onset_*	*M_inter_*	*M_intra_*
1	2	.54	.31	43.14	21.76	12.51	8.33
2	2	.57	.44	46.78	23.64	9.76	2.91
3	2	.53	.51	50.19	28.19	14.95	1.72
4	2	.16	.46	56.69	32.67	14.23	1.84
5	2	.33	.64	53.72	29.75	17.77	1.33
6	2	.30	.41	58.18	25.62	24.71	2.47
7	2	.39	.49	53.44	24.95	20.20	4.06
8	2	.51	.48	47.23	24.45	18.22	1.33
9	2	.13	.49	52.77	22.96	24.24	2.66
10	2	.15	.51	54.15	33.53	11.96	1.87
11	2	.59	.63	39.46	17.28	14.92	1.43
12	3	.32	.52	50.60	17.82	14.36	1.81
13	3	.16	.58	61.33	31.18	15.42	2.03
14	3	.46	.59	53.83	17.24	15.26	0.77
15	3	.43	.64	50.45	15.19	13.71	0.86
16	3	.35	.69	50.24	13.76	16.69	1.52
17	3	.39	.79	48.30	12.61	15.90	1.36
18	3	.61	.63	47.01	18.16	10.83	1.18
19	4	.21	.49	55.03	14.07	13.20	1.74
20	4	.30	.60	53.01	11.22	13.38	2.54
21	4	.26	.67	55.05	12.25	14.08	1.46
22	5	.21	.53	57.86	12.87	13.35	1.42
Mean	2.73	.36	.55	51.79	20.96	14.30	1.61

Note. Rules = number of rules contained in the item, *P* = item difficulty, *r_i_*_(*t-i*)_ = item-rest correlation, *M_ToT_* = mean time on task, *M_onset_* = mean onset time, *M_inter_* = mean interrule time, *M_intra_* = mean intrarule time.

**Table 2:** Characteristics of the two clusters.

Cluster	*n*	*M_score_* (*SD*)	*M_onset_* (*SD*)	*M_inter_* (*SD*)	*M_intra_* (*SD*)	*M_ToT_* (*SD*)
1	87	2.34 (1.63)	22.82 (9.44)	10.21 (4.38)	1.51 (0.64)	46.37 (15.95)
2	111	12.24 (4.39)	19.50 (6.70)	17.50 (4.98)	1.69 (0.76)	56.04 (11.21)

Note. *n* = sample size, *M_score_* = mean test score, *M_ToT_* = mean time on task, *M_onset_* = mean onset time, *M_inter_* = mean interrule time, *M_intra_* = mean intrarule time, *SD* = standard deviation.

**Table 3:** Accelerating effect of the log file times and its correlation to the test performance.

Measure	Δ*M*	*t*	*p*_Δ*M*_	*d*	*r*	*p_r_*
Structured Cluster (*n* = 111)						
ToT	−2.72	–	–	–	−.13	.178
Onset	13.71	18.87	<.001	1.66	−.14	.138
Inter	3.06	3.85	<.001	0.41	.31	<.001
Intra	0.81	3.73	<.001	0.47	−.01	.934
Unstructured Cluster (*n* = 87)						
ToT	2.23	–	–	–	.04	.682
Onset	12.44	11.64	<.001	1.17	.18	.097
Inter	1.29	1.65	.051	0.20	.06	.590
Intra	0.90	3.58	<.001	0.49	.02	.835

Note. ToT = time on task, Onset = onset times, Inter = interrule times, Intra = intrarule times, Δ*M* = mean difference of the times in the first and the last third of the test (accelerating effect), *t* = t statistic of the accelerating effect, *p*_Δ*M*_ = *p*-value of the accelerating effect, *d* = Cohen’s *d* effect size of the accelerating effect, *r* = correlation between the accelerating effect and the test score, *p_r_* = *p*-value of the correlation.

**Table 4:** Results of the hierarchical regression of the DESIGMA score on the time on task and the log file times.

Statistics	ToT Only		ToT and LFT				
	ToT	Total	ToT	Onset	Inter	Intra	Total
β	−0.22	–	0.35	−0.53	−0.63	−0.41	–
*t* or *F*	−7.11	50.58	2.29	−5.57	−4.95	−5.65	33.73
*df*	109	1, 109	106	106	106	106	4, 106
*p*	<.001	<.001	.024	<.001	<.001	<.001	<.001
*R* ^2^	31.70%	31.70%	2.18%	12.87%	10.19%	13.25%	56.00%
Δ*R*^2^	–	–	–	–	–	–	24.30%

Note. ToT = time on task, Onset = onset times, Inter = interrule times, Intra = intrarule times, β = beta regression coefficient, t = t statistic, F = F-statistic, *p* = *p*-value, R^2^ = (unique) determination coefficient, ΔR^2^ = change in variance explanation between the models.

**Table 5:** IAP effects on response times from a linear mixed-effects model.

Predictor	ToT		Interrule Times	
	*b*	*p*	*b*	*p*
Global IAP	14.84	.008	1.52	.615
Current IAP	−0.37	.627	0.08	.887

Note. IAP = incomplete attempt propensity, ToT = time on task.

## Data Availability

The original data presented in the study and the *R* script for statistical analysis are openly available on the *Open Science Framework* (OSF) at https://osf.io/kgdx9/?view_only=a11e56e591e34f74be2dafea55c56f39 (accessed on 19 May 2025).

## Abbreviations

The following abbreviations are used in this manuscript:

IAP	Incomplete Attempt Propensity
LFTs	Log File Times
RAPM	Raven’s Advanced Progressive Matrices
RtD	Response to Difficulty
ToT	Time on Task
